# Plasma cfDNA and peripheral blood gDNA provide complementary information for molecular monitoring in myeloproliferative neoplasms

**DOI:** 10.3389/fonc.2026.1771587

**Published:** 2026-03-27

**Authors:** Xiaotong Ma, Can Yang, Yusi Duan, Huixuan Zhang, Xuemei Tang, Qingyun Zhang, Wenjiao Qin, Wenhao Zhang, Gusheng Tang, Ming Guan, Zhiyuan Wu

**Affiliations:** 1 Department of Laboratory Medicine, Huashan Hospital, Fudan University, Shanghai, China; 2 Central Laboratory, Huashan Hospital, Fudan University, Shanghai, China; 3 Department of Hematology, Huashan Hospital, Fudan University, Shanghai, China; 4 Department of Hematology, the First Affiliated Hospital of Naval Medical University, Shanghai, China

**Keywords:** bone marrow, cell-free DNA, liquid biopsy, lymphocyte percentage, molecular monitoring, myeloproliferative neoplasms, treatment response

## Abstract

**Background:**

Molecular monitoring of Philadelphia-negative myeloproliferative neoplasms (MPN) relies on genomic DNA (gDNA) from peripheral blood (PB). As variant allele frequency (VAF) reduction correlates with clinical outcomes, optimizing monitoring strategies has become important. However, whether gDNA accurately reflects bone marrow (BM) clonal dynamics and whether cell-free DNA (cfDNA) provides complementary information remains undefined.

**Methods:**

We compared cfDNA and PB gDNA using methylation-based deconvolution, a cell line-derived xenograft (CDX) model, and paired clinical samples from two independent MPN cohorts (technical, n=7 with matched BM; validation, n=40 with longitudinal monitoring).

**Results:**

Methylation deconvolution showed an enrichment of BM-derived DNA in cfDNA (47.4% vs 18.9%, *P* = 0.035), providing exploratory evidence of its origin. The CDX model indicated that cfDNA serves as a superior surrogate for BM clonal burden, whereas gDNA underestimated it (*P* < 0.001). Clinically, mutation VAF showed high concordance (r=0.887, *P* < 0.001), yet cfDNA detected higher VAF (+22.6% mean advantage). Gene-specific analysis revealed *CALR* mutations had the 59.3% cfDNA advantage (*P* = 0.009). During treatment monitoring, cfDNA showed a trend toward higher sensitivity for resistance detection (25.6% vs. 17.9%), whereas gDNA appeared to be more sensitive for response confirmation (23.1% vs. 15.4%). Lymphocyte percentage emerged as a novel predictor of cfDNA advantage (r = 0.765, *P* = 0.045 in technical; r = 0.509, *P* < 0.001 in validation).

**Conclusions:**

While constrained by sample size and specific treatment contexts, our observations suggest that cfDNA and gDNA provide complementary value for MPN monitoring. Supported by preclinical models and technical analysis, cfDNA more effectively reflects BM clonal burden, while longitudinal observations suggest its capacity to track treatment-related clonal dynamics. In contrast, gDNA is more informative in confirming treatment response. The lymphocyte percentage predicts cfDNA utility, enabling rational test selection and a practical framework for optimizing MPN management.

## 1 Introduction

Philadelphia-negative myeloproliferative neoplasms (MPN), including polycythemia vera (PV), essential thrombocythemia (ET), and primary myelofibrosis (PMF), are clonal hematopoietic disorders characterized by excessive proliferation of mature hematopoietic cells from one or more myeloid lineages (1). MPN pathogenesis is driven by somatic mutations in *JAK2*, *CALR*, and *MPL* that constitutively activate the JAK/STAT signaling pathway (2–4). Additional mutations affecting epigenetic regulation (*ASXL1*, *DNMT3A*, *EZH2*, *IDH1/2*, *TET2*), RNA splicing (*SF3B1, SRSF2*), signal transduction (*NRAS/KRAS*), and DNA damage repair (*TP53*) further influence clonal dynamics and disease progression (5–8). Molecular profiling is essential for diagnosis and increasingly guides therapeutic decisions, with variant allele frequency (VAF) serving as a surrogate for clonal burden. Emerging evidence demonstrates that molecular response (significant VAF reduction) correlates with improved event-free survival and reduced risk of thrombosis, hemorrhage, and transformation (9–11). As disease modification becomes a realistic therapeutic goal in MPN, optimizing molecular monitoring strategies has become clinically imperative.

Current clinical practice relies predominantly on genomic DNA (gDNA) extracted from peripheral blood (PB) leukocytes for molecular monitoring. However, PB gDNA represents only the mature nucleated circulating cell population, excluding contributions from enucleated red blood cells and platelets, both of which carry the clonal mutation in MPN (12). Since MPN originates when a driver mutation is acquired by a hematopoietic stem cell (HSC) residing in the bone marrow (BM) (13, 14), PB gDNA may underestimate the true BM clonal expansion, particularly during early disease progression or treatment response. This limitation is compounded in specific clinical scenarios: patients with cytopenias may show diluted clonal signals; those with hypercellular marrows may exhibit discordant peripheral and marrow burdens; and emerging treatment resistance can be masked by the slow turnover of mature circulating cells (11). The invasive nature of BM biopsy limits dynamic monitoring, creating a clinical need for alternative strategies that more directly reflect marrow clonal dynamics.

Circulating cell-free DNA (cfDNA) comprises short DNA fragments released predominantly through apoptosis or necrosis. cfDNA has emerged as a powerful tool for non-invasive molecular monitoring in solid tumors (15–17). Importantly, recent reports have revealed that cfDNA originates predominantly from bone marrow-resident cells, with megakaryocytes alone contributing approximately 26% of plasma cfDNA in healthy individuals (18, 19). This proportion increases in conditions characterized by megakaryocyte expansion (ET), suggesting that cfDNA may provide a more direct window into marrow clonal dynamics than conventional PB gDNA. In myelodysplastic syndromes and acute myeloid leukemia (AML), cfDNA recapitulates clonal evolutionary trajectories consistent with BM samples and is regarded as a promising alternative method (20–22). Garcia-Gisbert and colleagues demonstrated that cfDNA analysis yields comparable mutational profiles with higher VAF detection (23). However, the biological basis for these differences, their clinical implications for treatment monitoring, and clinical parameters that predict cfDNA utility remain undefined.

These observations led us to hypothesize that cfDNA and gDNA capture complementary rather than redundant aspects of MPN biology. Here, we present a comprehensive investigation comparing plasma cfDNA versus PB gDNA for molecular monitoring in MPN. Through integrated analysis of methylation-based deconvolution and a cell line-derived xenograft (CDX) model, our study explores the biological origin of cfDNA and its relationship to BM-derived clonal signals. We further assess the clinical utility of cfDNA and PB gDNA in two MPN cohorts. Importantly, we identify lymphocyte percentage as a novel clinical predictor of cfDNA advantage, providing a simple, readily available parameter to guide the selection of analyses. Together, these analyses motivate a framework for the complementary, rather than competitive, application of cfDNA and gDNA in MPN monitoring.

## 2 Materials and methods

### 2.1 Patients and sample collection

This study enrolled patients with MPN between March 2023 and July 2025. BM and matched PB samples were obtained from 7 patients treated at the First Affiliated Hospital of Naval Medical University (Approval No. CHEC2022-076) (technical cohort: designed to establish cfDNA equivalence to BM). A total of 79 PB samples were collected from 40 additional patients at the Department of Hematology, Huashan Hospital, Fudan University (Approval No. 2022-1082) for longitudinal monitoring (validation cohort: designed to assess clinical utility during treatment). All diagnoses were established according to the fifth edition of the World Health Organization classification of hematolymphoid tumors. Complete blood count parameters, including white blood cell count (WBC), red blood cell count (RBC), hemoglobin (HGB), hematocrit (HCT), platelet count (PLT), and differential counts (covering percentages of neutrophils, lymphocytes, monocytes, eosinophils, and basophils) were collected. Treatment modalities and disease status were obtained along with clinical samples. The study was approved by the institutional review boards of both participating centers and conducted in accordance with the Declaration of Helsinki.

BM aspirates and PB samples were collected in K^3^EDTA tubes and processed within 6 hours. Samples were centrifuged at 3000 rpm for 10 minutes to separate plasma, which was stored at −80°C. cfDNA was extracted from plasma using the Quick-cfDNA Serum & Plasma Kit (Zymo Research). Genomic DNA from PB or BM was isolated using the QIAamp DNA Blood Mini Kit (Qiagen). DNA concentrations were quantified using a Qubit Fluorometer with the dsDNA High Sensitivity Assay Kit (Thermo Fisher Scientific).

### 2.2 Next-generation sequencing analysis

Targeted next-generation sequencing was performed using a custom panel covering 42 myeloid-associated genes: *ABL1, ASXL1, BCORL1, CALR, CBL, CSF3R, CUX1, DDX41, DNMT3A, EP300, ETV6, ETNK1, EZH2, FLT3, GATA2, IDH1, IDH2, IKZF1, JAK2, JAK3, KIT, KMT2D, KRAS, MPL, NF1, NPM1, NRAS, PAX5, PHF6, PTPN11, RBBP6, RUNX1, SETBP1, SF3B1, SH2B3, SRSF2, SUZ12, TET2, TP53, U2AF1, WT1*, and *ZRSR2*.

For cfDNA, libraries were prepared using a unique molecular identifier (UMI)-based workflow with an initial input of 30 ng. Sequencing was conducted on the NovaSeq S4 platform (Illumina), achieving an average sequencing depth exceeding 1000×. Over 94% of the targeted regions achieved a molecular coverage of at least 50 unique molecules after deduplication. In parallel, gDNA libraries were prepared from 200 ng input and sequenced to a mean depth of 500×. Target enrichment specificity and mapping efficiency exceeding 99% for both cfDNA and gDNA libraries.

To ensure analytical consistency, paired-end reads were aligned to the GRCh37 reference genome. For cfDNA, variant calling was performed on UMI-collapsed consensus reads. Subsequent filtering removed low-complexity regions, blacklisted sites, and common germline polymorphisms indexed in public databases, including 1000 Genomes, ClinVar, and ExAC.

Variants with VAF >2% were considered for analysis, representing a conservative reporting threshold applied consistently across sample types (23).

### 2.3 Cell line-derived xenograft model

HEL and HEK-293T cells were obtained from the Cell Bank of the Chinese Academy of Sciences. To establish the CDX model, male NSG mice (6–8 weeks, n=24) were used, with six receiving intravenous injection of PBS as negative controls. The remaining 18 mice received intravenous injection of 3 × 10^6^ luciferase-labeled HEL cells. After a 2-week engraftment period, HEL-engrafted mice were randomized into HEL-burden (HEL; vehicle), hydroxyurea-treated (HU; 100 mg/kg/day), or ruxolitinib-treated (RUX; 50 mg/kg/day) groups (n = 6 per group) and treated for four weeks. Engraftment was confirmed using *in vivo* bioluminescence imaging. BM, PB, and spleen samples were collected for subsequent analyses. All animal experiments were conducted in accordance with the guidelines of the Animal Ethics Committee of Fudan University (Fudan University; Protocol 2024-HSYY-156).

### 2.4 Droplet digital PCR analysis

For samples derived from the CDX model, genomic DNA from BM and PB, as well as cfDNA, was analyzed using droplet digital PCR (ddPCR) to quantify *JAK2*^V617F^ allele frequency, with mouse *rpp30* serving as the internal reference gene. ddPCR reactions were performed using the QX200 AutoDG Droplet Digital PCR System (Bio-Rad). Data analysis was performed with QuantaSoft software using Poisson statistics.

### 2.5 Methylation profiling and deconvolution analysis

Genome-wide DNA methylation profiling was performed on paired PB cfDNA and gDNA samples from 3 patients with newly diagnosed PV. Bisulfite-converted DNA was hybridized to the Infinium MethylationEPIC 850k BeadChip (Illumina). We constructed a reference methylation atlas comprising 582 CpG sites that distinguish BM from PB using published data from healthy individuals. Reference-based deconvolution was applied using an algorithm developed by Moss et al. to assess the cellular origin and compositional differences between cfDNA and gDNA (24). Details are provided in **Supplementary Methods**.

### 2.6 Statistical analysis

All statistical analyses were performed using GraphPad Prism version 9.5, R version 4.3.0, and Python 3.9 with SciPy.Stats. Comparisons between continuous variables were performed using a t-test, and non-parametric comparisons were analyzed using the Wilcoxon signed-rank test or Mann-Whitney test. Categorical variables were assessed using χ² or Fisher’s exact tests. Pearson or Spearman correlation coefficients were used to evaluate associations with clinical parameters, and linear regression was applied to model predictors of cfDNA performance. Effect size was calculated using Cohen’s d. A two-sided *P* < 0.05 was considered statistically significant, and *P* < 0.10 was interpreted as suggestive in exploratory analyses.

## 3 Results

### 3.1 cfDNA and gDNA sample distinct biological compartments

**Figure 1:**
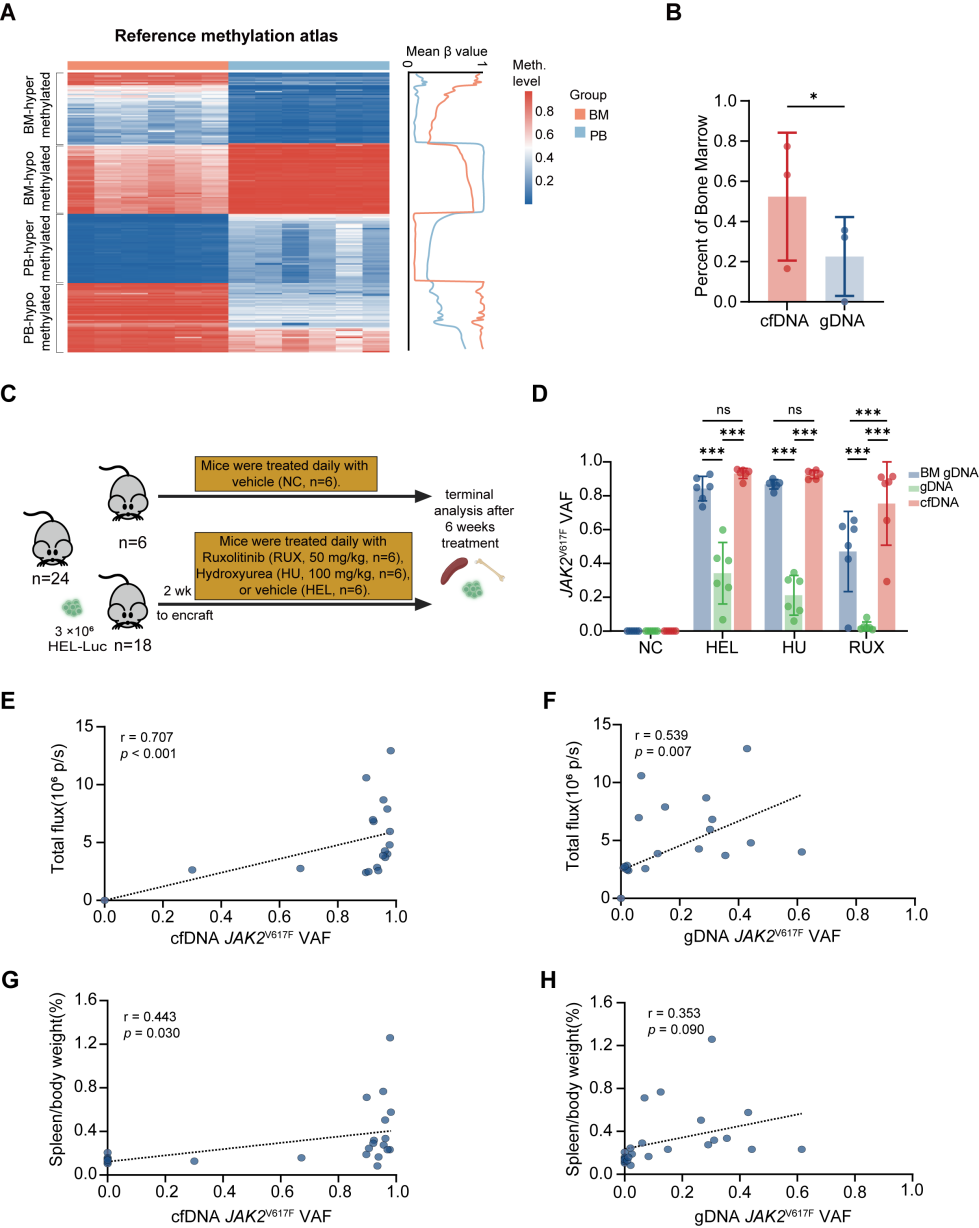
cfDNA and gDNA sample distinct biological compartments. **(A)** Reference methylation atlas constructed from 6 healthy BM and 6 healthy PB samples. The heatmap shows CpG methylation sites distinguishing BM from PB. **(B)** Proportion of BM contribution inferred from cfDNA and gDNA in MPN patients by methylation deconvolution. Bars show mean ± SD; *P < 0.05 by paired t-test. **(C)** Schematic of experimental workflow. NSG mice were intravenously injected with HEL-Luc cells, allowed to engraft for 2 weeks, and subsequently treated with vehicle control (HEL), hydroxyurea (HU), or ruxolitinib (RUX) for 4 weeks. **(D)** Quantification of *JAK2*^V617F^ VAF by ddPCR in BM gDNA, PB gDNA, and cfDNA across treatment groups. Bars show mean ± SD; ***P < 0.001 by paired t-test. **(E)** Correlation between cfDNA *JAK2*^V617F^ VAF and total bioluminescence signals (r = 0.707, *P* < 0.001). **(F)** Correlation between gDNA *JAK2*^V617F^ VAF and total bioluminescence signals (r = 0.539, *P* = 0.007). **(G)** Correlation between cfDNA *JAK2*^V617F^ VAF and spleen/body weight ratio (r = 0.443, *P* = 0.030). **(H)** Correlation between gDNA *JAK2*^V617F^ VAF and spleen/body weight ratio (r = 0.353, *P* = 0.090).

To establish the biological basis for potential differences between cfDNA and gDNA, we first investigated their tissue origins using DNA methylation-based deconvolution. We obtained whole BM and whole PB methylation data from healthy individuals published in the Gene Expression Omnibus (GEO) and constructed a reference methylation atlas comprising 582 CpG sites that distinguish BM from PB (**Figure 1A**; **Supplementary Table 1**). This atlas was computationally validated using a deconvolution algorithm and reliably detected BM components at levels as low as 1% and PB components at levels as low as 2.86% (**Supplementary Figure 1**).

We then collected paired gDNA and cfDNA samples from 3 newly diagnosed PV patients, obtained methylation profiles using Illumina EPIC 850K arrays, and performed deconvolution analysis. In this exploratory analysis with a limited sample size (n=3), we observed that 47.4% (± 30.8% SD) of cfDNA was estimated to be derived from BM, compared with 18.9% (± 16.5% SD) in gDNA (*P* = 0.035; **Figure 1B**). While the large SD reflects substantial inter-individual heterogeneity, this 2.5-fold enrichment of BM-derived DNA suggests a possible mechanistic basis is that cfDNA may predominantly originate from the BM. Given the limited sample size (n=3), these findings are intended to preliminarily illustrate the potential compositional differences between cfDNA and gDNA rather than provide a definitive foundation.

To experimentally validate the methylation findings, we generated a HEL-based CDX model (**Figure 1C**; **Supplementary Figure 2**). NSG mice received intravenous injection of HEL-luc cells and were randomized to vehicle control, hydroxyurea, or ruxolitinib treatment. Whole-body bioluminescence imaging confirmed systemic infiltration, with ruxolitinib markedly reducing total photon intensity (*P* = 0.009; **Supplementary Figure 2A**).

Critically, we quantified *JAK2*^V617F^ VAF using ddPCR across sample types (**Figure 1D**). In the HEL and HU cohorts, cfDNA VAF was comparable to BM values, whereas PB gDNA consistently and markedly underestimated BM clonal burden across all three treatment conditions (*P* < 0.001). In the RUX cohort, cfDNA VAF was slightly higher than BM (*P* < 0.001), potentially reflecting increased cfDNA release induced by RUX treatment. To provide independent biological validation, we performed correlation analyses between VAF and systemic disease indicators. Across all treatment groups, cfDNA VAF demonstrated a substantial correlation with total bioluminescence signals (r = 0.707, *P* < 0.001; **Figure 1E**), while the explanatory power of gDNA was notably lower (r = 0.539, *P* = 0.007; **Figure 1F**). Regarding spleen indices (spleen to body weight ratio), cfDNA exhibited a detectable, consistent association with disease burden (r = 0.443, *P* = 0.030; **Figure 1G**), whereas the correlation observed for gDNA did not reach statistical significance (r = 0.353, *P* = 0.090; **Figure 1H**). Although this controlled CDX model does not fully reflect the complex mutational heterogeneity of MPN, these multiparametric correlations suggest that cfDNA may serve as a more representative surrogate for BM clonal status compared with PB gDNA.

### 3.2 Clinical validation reveals high concordance with predictable differences

Having established that cfDNA and gDNA sample different biological compartments, we next examined whether this translates into clinically meaningful detection differences in MPN patients. We evaluated cfDNA and gDNA performance in two complementary cohorts. The technical cohort (n=7) included matched BM samples to preliminarily assess whether cfDNA approximates BM values. The validation cohort (n=40) provided longitudinal paired cfDNA and PB gDNA to assess clinical utility during treatment monitoring.

**Figure 2:**
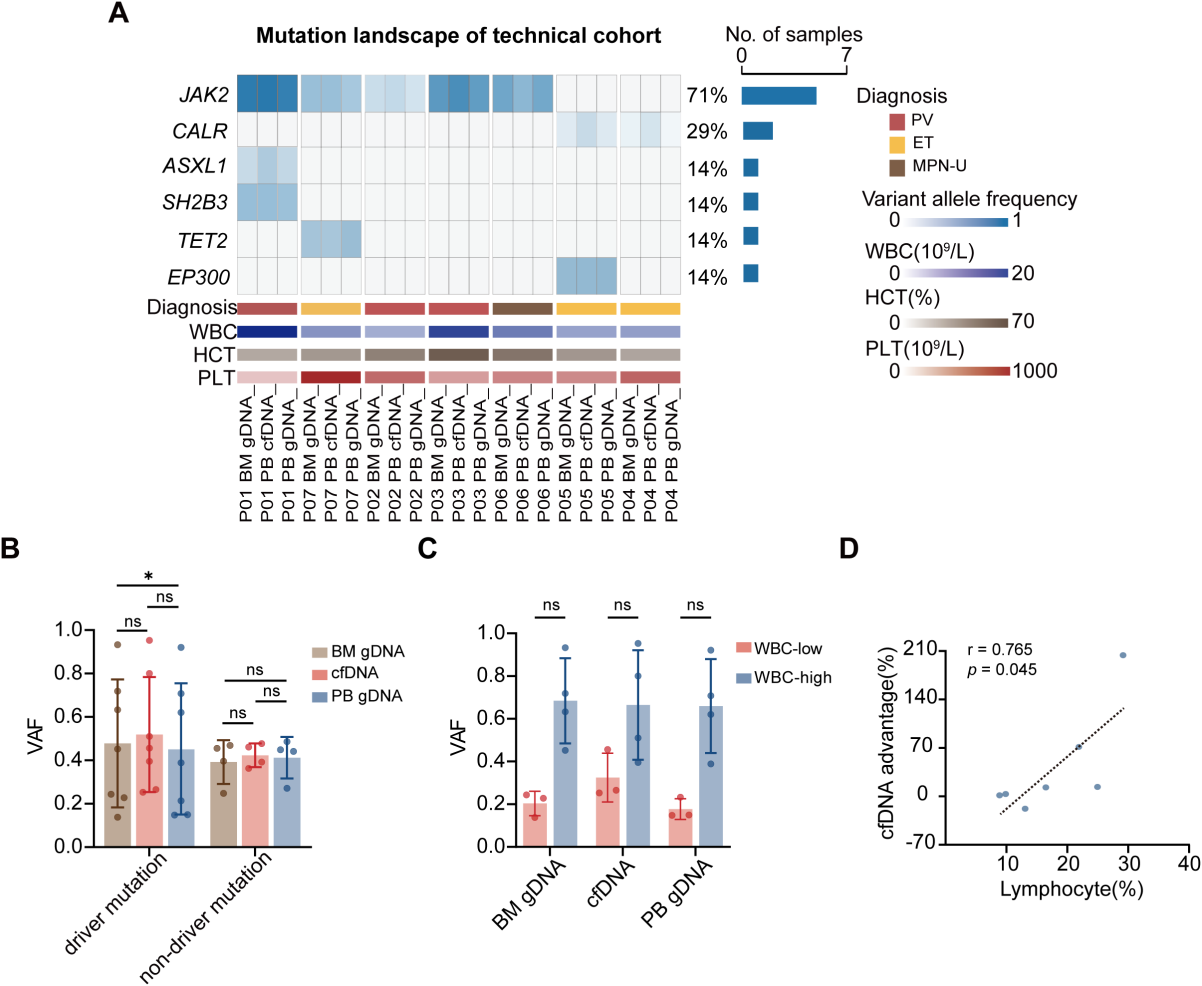
Technical cohort validation with matched BM samples. **(A)** Mutation landscape of the technical cohort (n = 7) across paired BM, plasma cfDNA, and PB gDNA samples. Each column represents one patient, and each row represents mutations grouped by gene. VAF is displayed using a continuous color scale. **(B)** VAF comparison of driver and non-driver mutations across BM gDNA, cfDNA, and PB gDNA. Bars show mean ± SD; *P < 0.05 by paired t-test. **(C)** Impact of WBC count on VAF measurements across BM gDNA, cfDNA, and PB gDNA. *P*-value by independent-samples t-test. **(D)** Correlation between lymphocyte percentage and cfDNA advantage showing the predictive relationship (r = 0.765, *P* = 0.045).

The technical cohort comprised 7 patients (3 PV, 3 ET, 1 MPN-U). Mean age was 70 years (range 60-80), with 29% male. Treatment regimens included hydroxyurea (29%), symptomatic treatment (29%), interferon-alpha (14%), ruxolitinib (14%), or aspirin (14%). Paired BM gDNA, PB gDNA, and cfDNA samples were collected to preliminarily compare cfDNA and gDNA for their equivalence in reflecting BM clonal burden. Targeted NGS analysis of 11 somatic mutations was performed. Driver mutations in *JAK2* (n = 5) and *CALR* (n = 2), as well as non-driver mutations in *ASXL1*, *SH2B3*, *TET2*, and *EP300* (one each), were consistently detected in both PB gDNA and cfDNA whenever present in the BM (**Figure 2A**; **Supplementary Table 2**). For driver mutations, there was no significant difference between BM gDNA and cfDNA, whereas PB gDNA VAF was modestly lower than BM gDNA (*P* = 0.031). For non-driver mutations, no significant differences were observed among PB gDNA, cfDNA, and BM samples (**Figure 2B**). Meanwhile, we observed a potential detection advantage of cfDNA over gDNA for certain genes (**Supplementary Figure 3**). Collectively, considering the small sample size (n=7), these results suggest a technical concordance of cfDNA with BM for driver mutations detection. The comparative performance of cfDNA and gDNA was then validated in the validation cohort.

**Figure 3:**
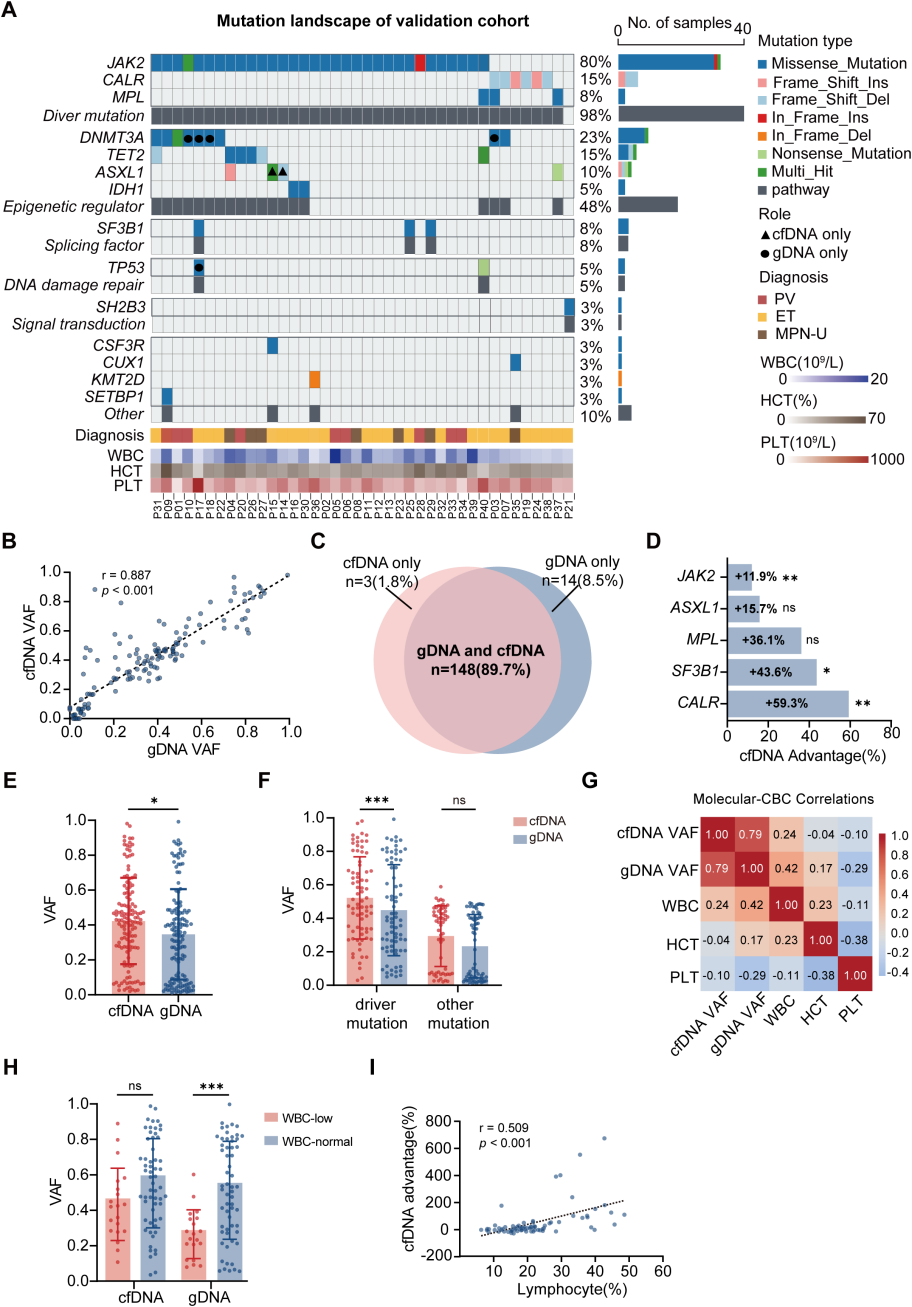
Validation cohort confirms gene-specific cfDNA advantage. **(A)** Mutation landscape of validation cohort (n=40). Each column represents one patient; rows show mutations grouped by function. Mutations detected exclusively in a single analyte are indicated by distinct symbols (triangle for cfDNA-only; square for gDNA-only). **(B)** Correlation of VAF between cfDNA and gDNA (r = 0.887, *P* < 0.001). **(C)** Venn diagram of concordant and discordant mutations between methods. **(D)** Gene-specific detection rate comparison showing cfDNA advantages for *CALR* (59.3%), *SF3B1* (43.6%), *MPL* (36.1%), *ASXL1* (15.7%), and *JAK2* (11.9%). *P < 0.05, **P < 0.01 by paired t-test. **(E)** VAF comparison of all mutations between cfDNA and PB gDNA. Bars show mean ± SD; *P < 0.05, by independent-samples t-test. **(F)** VAF comparison of driver mutations and other mutations between cfDNA and PB gDNA. Bars show mean ± SD; ***P < 0.001 by independent-samples t-test. **(G)** Correlation heatmap showing cfDNA independence from peripheral blood parameters. **(H)** Impact of WBC count on VAF measurements across cfDNA and PB gDNA. Bars show mean ± SD; ***P < 0.001 by independent-samples t-test. **(I)** Correlation between lymphocyte percentage and cfDNA advantage showing the predictive relationship (r = 0.509, *P* < 0.001).

The validation cohort comprised 40 patients (9 PV, 24 ET, 7 MPN-U). Median age was 66 years (range 43-87), with 40% male. Treatment regimens included hydroxyurea monotherapy (70%), interferon-alpha (13%), combination therapy (8%), ruxolitinib (3%), or aspirin alone (5%). Median follow-up was 3.7 years (IQR 2.1-4.9) (**Table 1**). Paired cfDNA and gDNA samples from two longitudinal time points were collected to compare differences in mutational detection and clonal dynamics. The mutational landscape was highly concordant between cfDNA and gDNA (**Figure 3A**; **Supplementary Table 3**). The phenotypic driver mutations of MPN were present in 98% of patients:*JAK2* (80%), *CALR* (15%), and *MPL* (8%). The mostfrequent non-driver mutations affected epigenetic regulators: *DNMT3A* (23%), *TET2* (15%), *ASXL1* (10%), and *IDH1* (5%). Additional alterations included splicing factor gene *SF3B1* (8%), DNA damage repair gene *TP53* (5%), signaling gene *SH2B3* (3%), and other myeloid-related mutations, including *CSF3R*, *CUX1*, *KMT2D*, and *SETBP1* (10%). Despite the high overall concordance in mutational profiles, gDNA and cfDNA showed different detection patterns: gDNA detected more *DNMT3A* mutations, while cfDNA for *ASXL1* mutations (**Supplementary Table 4**).

**Table 1:** Patient characteristics.

Characteristic	Technical cohort (n=7)	Validation cohort (n=40)
Patient-related variables		
Female	5 (71%)	24 (60%)
Male	2 (29%)	16 (40%)
Age, median (range), years	70 (60–80)	66 (43–87)
Disease subtype		
Polycythemia Vera	3 (43%)	9 (23%)
Essential Thrombocythemia	3 (43%)	24 (60%)
Primary myelofibrosis	0 (0%)	0 (0%)
MPN-U/Other	1 (14%)	7 (18%)
Driver mutation		
*JAK2*	5 (71%)	32 (80%)*
*CALR*	2 (29%)	6 (15%)
*MPL*	0 (0%)	3 (8%)
Triple negative	0 (0%)	1 (3%)
Disease-related variables		
WBC, ×10^9^/L, median (range)	16(7.8-46.4)	11 (2.9-79.8)
Hematocrit, vol %	46 (34.0-64)	40 (19–68)
Hemoglobin, g/L	146 (99-178)	131 (58-206)
Platelets, ×10^9^/L	625 (283-1101)	496 (40-2166)
Treatment		
Hydroxyurea	2 (29%)	28 (70%)
Interferon	1 (14%)	5 (13%)
Ruxolitinib	1 (14%)	1 (3%)
IFN+HU	0 (0%)	3 (8%)
Symptomatic treatment/Aspirin	3 (43%)	2 (5%)

Data are presented as n (%) or median (range) unless otherwise indicated.Coexisting *MPL/JAK2* mutations were detected in 1 patient, and CALR/MPL mutations in 1 patient.

Mutation VAF showed strong concordance between gDNA and cfDNA (r=0.887, *P* < 0.001; **Figure 3B**). Of 165 somatic mutations detected, 148 (89.7%) were identified by both methods, 3 (1.8%) were detected only by cfDNA, and 14 (8.5%) only by gDNA (**Figure 3C**). Gene-specific analysis confirmed cfDNA superiority for *CALR* (59.3% mean advantage, *P* = 0.009, n=12), *SF3B1* (43.6%, *P* = 0.031, n=6), and *JAK2* (11.9%, *P* = 0.002, n=62). *MPL* (36.1%, *P* = 0.063, n=5) and *ASXL1* (15.7%, *P* = 0.160, n=10) showed trends toward cfDNA advantage that did not reach statistical significance (**Figure 3D**).

Overall VAF in cfDNA was higher than gDNA (0.424 vs 0.346, *P* = 0.011; **Figure 3E**). Stratification revealed cfDNA exhibited higher VAF specifically for driver mutations (0.522 vs 0.449, *P* < 0.001), whereas no significant difference was observed for non-driver mutations (**Figure 3F**). Subgroup analysis by driver mutations demonstrated that cfDNA VAF remained higher than gDNA VAF in patients harboring *JAK2* (0.557 vs 0.498, *P* = 0.002) and *CALR* (0.400 vs 0.251, *P* = 0.009) mutations, with a similar but non-significant trend observed in the *MPL* mutations (0.197 vs 0.144, *P* = 0.063; **Supplementary Figure 4**).

Together, these findings indicate that while cfDNA and gDNA share a broadly concordant mutational landscape, cfDNA shows superior reflection of driver mutation clonal burden. Given the absence of paired BM sampling, further studies incorporating paired samples in larger cohorts are required to evaluate the clinical validity of cfDNA as a surrogate for BM clonal burden. Gene-specific analysis highlighted a graded spectrum advantage, detailed in **Table 2**.

**Table 2:** Gene-specific cfDNA advantage in the validation cohort.

Gene	n	cfDNA Advantage*	P-value	Recommendation
*CALR*	12	59.30%	0.009	Prioritize cfDNA
*SF3B1*	6	43.60%	0.031	Prioritize cfDNA
*MPL*	5	36.10%	0.063	cfDNA preferred
*JAK2*	62	11.90%	0.002	Consider cfDNA
*ASXL1*	10	15.70%	0.160	Consider cfDNA
*TET2*	11	-0.33%	0.960	Consider gDNA
*DNMT3A*	20	-2.80%	0.757	Consider gDNA

*cfDNA advantage calculated as mean VAF difference (cfDNA-gDNA)/gDNA.

### 3.3 Hematologic parameters influence analyte-specific performance

We evaluated how hematologic parameters differentially influence cfDNA and gDNA measurements. In the technical cohort, stratification by a WBC threshold of 10×10^9^/L suggested a potential influence of leukocyte counts on VAF measurements from BM gDNA, cfDNA, and PB gDNA (*P* = 0.057, *P* = 0.114, and *P* = 0.057, respectively; **Figure 2C**). In the validation cohort, correlation analysis revealed differential dependencies on hematologic parameters (**Figure 3G**). gDNA VAF correlated moderately with WBC (r = 0.42), HCT (r = 0.17), and PLT (r = -0.29), whereas cfDNA VAF showed weaker correlations with these parameters (WBC: r = 0.24; HCT: r = -0.04; PLT: r = -0.1) (**Figure 3G**). Patients with lower WBC counts exhibited a significant reduction in PB gDNA VAF of driver mutations (*P* < 0.001), whereas cfDNA VAF remained relatively stable (*P* = 0.059; **Figure 3H**). Further stratification by WBC quartiles demonstrated that cfDNA VAF was significantly higher than gDNA VAF in patients with lower WBC counts (Q1 *P* = 0.003; Q2 *P* < 0.001), whereas this difference diminished at higher WBC levels (Q3 and Q4; **Supplementary Figure 5**). This pattern suggests that cfDNA measurements are less influenced by PB counts and may provide a more stable assessment of clonal burden under conditions of cytopenia.

The observation of gene-specific advantages of cfDNA, together with the differential influence of hematologic parameters on cfDNA and gDNA, raised the question of whether clinical parameters might predict which patients would benefit most from cfDNA testing. Integration of molecular data with comprehensive clinical parameters from the technical cohort revealed correlations that may guide clinical test selection. Lymphocyte percentage emerged as a potential predictor of cfDNA advantage (r = 0.765, *P* = 0.045; **Figure 2D**):


$$cfDNA_{\text{advantage}}=-93.64+7.549\times \text{LYMPH\%.}$$

This model suggests that for every 1% increase in lymphocyte percentage, cfDNA advantage increases by 7.5 percentage points. The association was subsequently validated in the validation cohort, demonstrating a similar linear trend with a weaker but highly significant association:


$$cfDNA_{\text{advantage}}=-84.48+6.167\times \text{LYMPH\% }(\text{r}=0.509,\;P<0.001)$$

(**Figure 3I**).

The biological basis for this correlation may relate to immune surveillance and the distinct origins of cfDNA and gDNA. A higher lymphocyte proportion may reflect a more active immune-mediated clearance process, thereby promoting the release of tumor-derived DNA into circulation, suggesting that the balance between myeloid and lymphoid compartments influences cfDNA composition. Meanwhile, PB gDNA is derived directly from circulating nucleated cells, and its VAF is susceptible to dilution by lymphocyte count relative to the mutant clone. In contrast, cfDNA is partly released from apoptotic malignant clones in the BM and therefore shows weaker correlations with CBC parameters, supporting its relative independence from PB cellular dynamics.

### 3.4 Longitudinal monitoring demonstrates complementary clinical utilities

**Figure 4:**
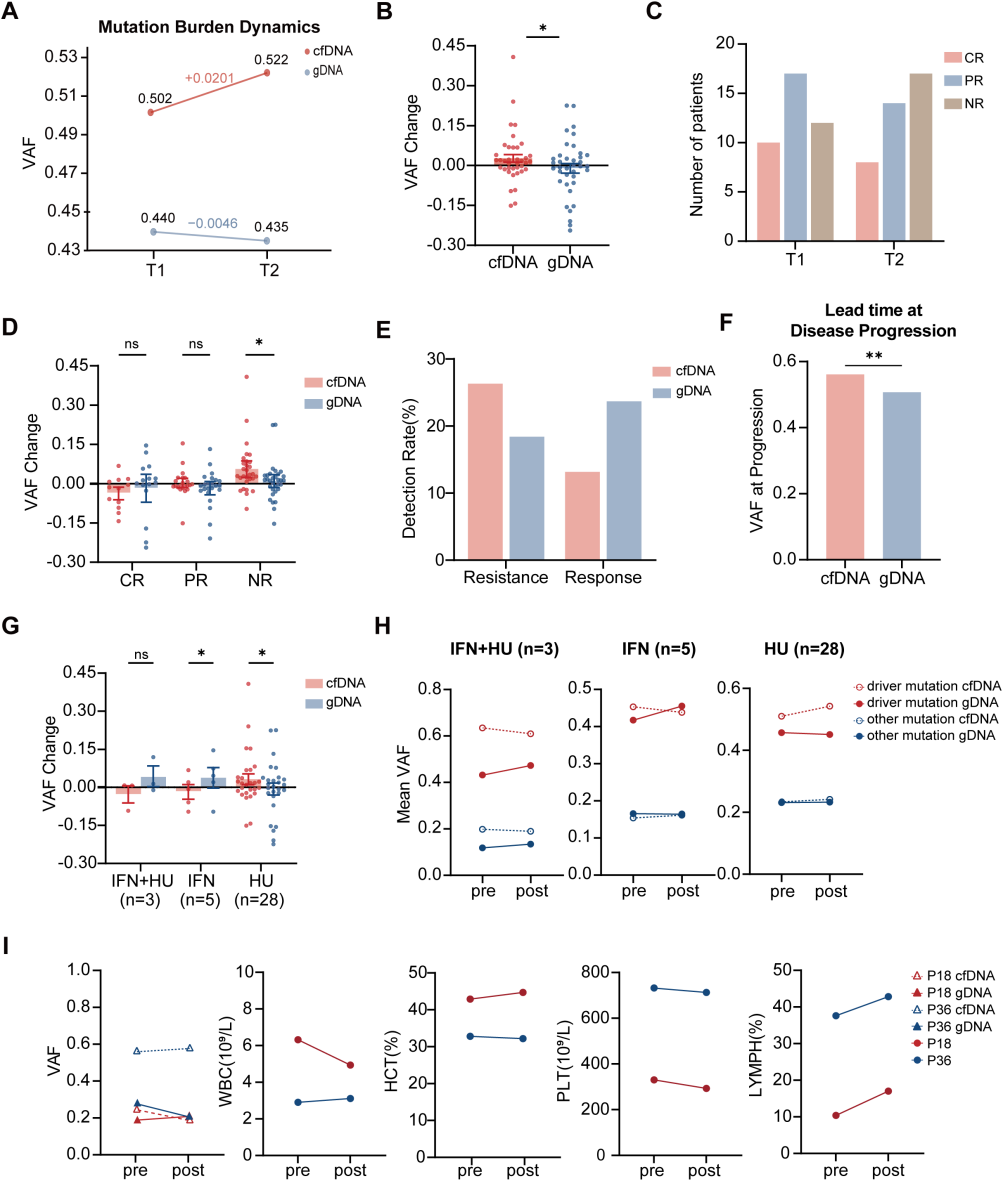
Longitudinal monitoring demonstrates complementary clinical utilities. **(A)** Mean VAF change between treatment timepoints T1 and T2 for cfDNA and gDNA. **(B)** Mean VAF change of driver mutations during treatment. Bars show mean ± SEM, *P < 0.05 by paired t-test. **(C)** Distribution of hematologic responses at T1 and T2. **(D)** VAF change stratified by hematologic response category. Bars show mean ± SEM, *P < 0.05 by paired t-test. **(E)** Classification of treatment resistance versus response. **(F)** VAF shift in patients with disease progression (≥10% increase). **P < 0.01 by independent-samples t-test. **(G)** Treatment-specific VAF changes showing divergent cfDNA and gDNA dynamics across IFN+HU, IFN, and HU regimens. Bars show mean ± SEM; *P < 0.05 by paired t-test. **(H)** Clonal evolution patterns of driver and non-driver mutations across treatment groups. **(I)** Integrated dynamic changes of clonal mutations and hematological parameters in two patients across treatment.

We evaluated cfDNA and gDNA performance in treatment monitoring by analyzing mutation burden between two treatment time points (T1 and T2). cfDNA showed an increase in mean VAF (0.502 to 0.522), whereas gDNA showed a slight decrease (0.440 to 0.435; **Figure 4A**). This discrepancy was more pronounced in driver mutations, where cfDNA VAF increased by a mean of 0.027, contrasting with the gDNA decrease (-0.009; *P* = 0.044; **Figure 4B**).

Hematologic responses were evaluated based on European LeukemiaNet criteria. At T1, 10 patients achieved a complete response (CR), 17 partial response (PR), and 12 no response (NR). By T2, the distribution shifted to 8 CR, 14 PR, and 17 NR, indicating worsening hematologic response overall (**Figure 4C**). In the CR and PR groups, cfDNA and gDNA showed comparable VAF changes, whereas in the NR group, cfDNA captured a significantly greater VAF increase than gDNA (*P* = 0.035; **Figure 4D**), indicating superior sensitivity for detecting treatment failure.

This divergence translated into clinically meaningful differences in the classification of treatment outcomes. Using a ≥ 5% VAF change threshold to define molecular response or resistance to distinguish clonal dynamics from smaller longitudinal fluctuations (**Supplementary Figure 6**), cfDNA identified a higher proportion of patients with treatment resistance compared with gDNA (25.6% vs. 17.9%), whereas gDNA classified a higher proportion of patients as achieving treatment response (23.1% vs. 15.4%; **Figure 4E**). When a ≥10% VAF increase defined disease progression, cfDNA exhibited a substantially larger VAF shift (0.561 vs 0.507, *P* = 0.004), indicating its advantage for identifying early progression (**Figure 4F**).

Treatment-specific analysis provided additional information regarding the complementary nature of these biomarkers. Subgroup analysis by treatment regimen suggested potentially divergent clonal dynamics (**Figure 4G**). In the IFN+HU combination group (n=3), cfDNA showed an overall reduction (-0.026) while gDNA displayed an increase (+0.041). In the IFN-treated group (n=5), cfDNA and gDNA showed discordant directional changes (cfDNA -0.015 vs gDNA +0.089, *P* = 0.035). In the HU-treated group (n=28), cfDNA detected a VAF increase (+0.038), whereas gDNA indicated a decline (-0.011, *P* = 0.024).

Detailed clonal profiling of 36 patients for both driver and non-driver mutations (**Figure 4H**) revealed markedly different evolution patterns. In the IFN+HU group, cfDNA showed VAF decreases in both mutation types, while gDNA showed increases. In the IFN group, cfDNA indicated a decrease in driver mutations but an increase in non-driver mutations, with gDNA showing the opposite pattern. In the HU group, cfDNA captured concurrent increases in both mutation types, while gDNA reflected a slight decrease in driver mutations alongside a slight increase in non-driver mutations. Individual patient clonal dynamics are provided in the **Supplementary Figure 7**. While these findings derive from small observational subsets, the consistent trends in directional divergence provide a mechanistic basis for the hypothesis that cfDNA and gDNA may reflect different processes during treatment.

To further contextualize these divergent patterns, we examined two representative patients with distinct treatment backgrounds. In an IFN-treated patient (P18), hematologic parameters showed concordant improvement, including a reduction in WBC (6.32 to 4.94 ×10^9^/L), relatively stable HCT (42.9% to 44.1%), and stable PLT counts (330 vs. 293 ×10^9^/L). In parallel, cfDNA VAF decreased by 5.9%, reaching the predefined molecular response threshold, whereas gDNA VAF showed a 2% increase. In contrast, in an HU-treated patient (P36), hematologic parameters did not indicate remission: although WBC (2.9 to 3.11 ×10^9^/L) remained low, PLT remained elevated (732 vs. 691 ×10^9^/L). In this context, cfDNA VAF remained largely stable, whereas gDNA VAF declined by 7.1%. Notably, this patient exhibited a high lymphocyte proportion (37.6% and 42.8%), suggesting that PB cellular composition may contribute to the gDNA VAF change (**Figure 4I**). Across both cases, cfDNA and gDNA displayed different longitudinal trajectories. These observations support that lymphocyte proportion may serve as a practical clinical parameter for selection and interpretation of cfDNA and gDNA in MPN monitoring.

## 4 Discussion

This study provides mechanistic and clinical evidence suggesting that plasma cfDNA and PB gDNA represent biologically distinct compartments with complementary utility in MPN monitoring. Our integrated approach, combining preclinical modeling: methylation-based deconvolution, and the CDX model, provided a biological basis indicating that cfDNA is enriched for BM-derived DNA and may more accurately reflect BM clonal burden than conventional gDNA analysis. Consistent with these preclinical insights, our observations across two independent patient cohorts demonstrated the potential clinical utility of cfDNA and identified lymphocyte percentage as a novel predictor of cfDNA advantage.

The biological basis for this differential performance of cfDNA and gDNA lies in tissue origin: although limited by sample size, our methylation deconvolution analysis observed that nearly half (47.4%) of cfDNA in MPN patients derives from BM, compared with only 18.9% of gDNA. This estimate aligns with recent evidence that megakaryocytes contribute approximately 26% of plasma cfDNA in healthy individuals, with increased contributions in thrombocytosis-associated conditions (19). Since MPN are characterized by clonal megakaryocyte expansion, the BM enrichment of cfDNA explains its superior detection of driver mutations affecting megakaryocyte biology. We also found that cfDNA showed a weaker correlation with PB counts (WBC, HCT, PLT) compared to gDNA. While mutation burden is traditionally associated with WBC counts (25, 26), we observed that gDNA VAF declined significantly in patients with lower WBC (*P* < 0.001), whereas cfDNA VAF remained relatively stable (*P* = 0.059). This divergence may reflect the impact of the balance between myeloid and lymphoid compartments on gDNA measurements: in clinical contexts characterized by shifts in PB cell composition, a higher lymphocyte proportion can dilute the circulating myeloid clone in PB gDNA, leading to an underestimation of the BM clonal burden. In contrast, since cfDNA contains a higher proportion of BM-derived components, it remains independent of peripheral cellular fluctuations.

Our CDX model provided controlled experimental validation: cfDNA VAF closely matched BM values in HEL and HU cohorts, whereas PB gDNA consistently underestimated BM clone burden. Consistent with this, cfDNA VAF also demonstrated stronger correlations with objective measures of systemic tumor burden. In the RUX cohort, cfDNA VAF slightly exceeded BM VAF, likely reflecting increased release of mutant DNA associated with ruxolitinib-induced apoptosis (27–29). This highlights the importance of considering treatment timing when interpreting cfDNA measurements, as early therapeutic effects may transiently influence cfDNA levels. These findings translated directly to clinical observations: in our technical cohort, cfDNA exhibited no significant difference from BM for driver mutations, while gDNA VAF was slightly lower.

Despite high concordance in mutational profiles (r=0.887, *P* < 0.001), cfDNA demonstrated higher VAF for driver mutations, consistent with the previous reports (23). Discrepancies were observed for a subset of mutations. Mutations detected exclusively in PB gDNA were primarily *DNMT3A*, whereas those detected exclusively in cfDNA were mainly *ASXL1*. Although *DNMT3A* and *ASXL1* are both commonly implicated in clonal hematopoiesis, they exert fundamentally different effects on HSC biology and evolution (30). *DNMT3A* affects HSC by enhancing self-renewal, resulting in slow, stable clonal expansion that exists in mature PB cells over time (31). In contrast, *ASXL1* impairs HSC function and is associated with increased cellular turnover (32, 33), which may lead to enhanced release of mutant DNA into the circulation. Notably, *ASXL1* was identified only in cfDNA in a patient who subsequently progressed to secondary AML. These findings suggest that PB gDNA mainly reflects stable clones residing in the mature PB compartment, whereas cfDNA more effectively captures biologically active clonal dynamics. Specifically, gene-specific analysis revealed graded cfDNA advantage: *CALR* (59.3%), *SF3B1* (43.6%), *MPL* (36.1%), *ASXL1* (15.7%) and *JAK2* (11.9%). The *CALR* and *MPL* advantages likely reflect preferential shedding from expanded megakaryocyte compartments (34–36). This interpretation is consistent with Garcia-Gisbert et al., who observed that *MPL* mutations, also associated with megakaryocyte biology, showed higher VAF in cfDNA than in granulocytes (23). *SF3B1* and *ASXL1* advantages may reflect increased BM cell turnover associated with these high-risk mutations (37–39). Conversely, *TET2* and *DNMT3A* showed no cfDNA advantages, suggesting gDNA suffices for these mutations. Taken together, these findings highlight the potential of cfDNA for enabling early detection of emerging clones and monitoring transformation risk.

One of the clinically relevant findings was that cfDNA and gDNA capture different clonal trajectories during treatment. Specifically, cfDNA detected more resistance (25.6% vs 17.9%) and showed greater VAF increases in non-responders (*P* = 0.035), while gDNA better confirmed treatment response (23.1% vs 15.4%). These patterns were further illustrated by treatment-specific dynamics: IFNα-treated patients showed declining cfDNA with rising gDNA; HU-treated patients showed the opposite pattern (*P* = 0.024 and *P* = 0.035, respectively). These divergences may be explained by the distinct pharmacodynamics: IFNα is reported to activate quiescent mutated HSC, depleting the mutant clone and releasing cfDNA through stem cell apoptosis (40–42). Conversely, HU suppresses proliferating cells, rapidly reducing gDNA VAF while sparing quiescent stem cells that continue contributing to cfDNA (43–45). Consistent with prior reports that cfDNA reflects BM clonal burden and serves as an effective minimal residual disease surrogate (46, 47). Although these treatment-specific observations remain hypothesis-generating, given the cohort size, they provide a preliminary framework for understanding the complementary roles of cfDNA and gDNA in monitoring MPN.

The correlation between lymphocyte percentage and cfDNA advantage (r = 0.765, *P* = 0.045; r = 0.509, *P* < 0.001 in the validation cohort) suggests that immune-mediated tumor killing may enhance cfDNA release (48) or that lymphocyte predominance dilutes myeloid-derived gDNA. Either mechanism supports transforming a routine CBC parameter into a potential guide for test selection: using lymphocyte percentage >20% to identify patients who benefit most from cfDNA testing.

Consistent with Garcia-Gisbert et al., we observed a highly concordant mutational landscape between cfDNA and PB gDNA, with cfDNA displaying a modestly higher overall VAF (23). Beyond this overall concordance, our analyses indicate that the two analytes are not fully equivalent across clinical contexts. Specifically, gDNA-derived VAF were more susceptible to variation in PB counts, whereas cfDNA showed weaker correlations with hematologic parameters. Moreover, longitudinal analyses revealed that cfDNA and gDNA could display discordant clonal dynamics under different treatment settings, with cfDNA being able to capture molecular changes associated with treatment resistance under certain conditions.

Taken together, these findings support a complementary use of cfDNA and gDNA in MPN management. Compared with gDNA testing, cfDNA analysis requires deeper sequencing and more stringent pre-analytical handling, and therefore is not recommended to universally replace gDNA. Instead, a selective application that balances clinical utility and cost-effectiveness is required. PB gDNA remains the most practical option for routine monitoring of clinically stable patients and for tracking *DNMT3A*/*TET2* mutations. In contrast, cfDNA may be more informative for specific clinical utilities, including monitoring of *CALR*/*SF3B1*/*MPL*/*JAK2*/*ASXL1* mutations, early assessment of treatment resistance, and evaluation of patients with cytopenias or elevated lymphocyte percentages (>20%). Dual monitoring is best reserved for critical therapeutic decision points or cases of molecular-hematologic discordance.

Our study has several limitations. First, the methylation deconvolution analysis included only three patients, though results were statistically significant and mechanistically consistent with preclinical data. Similarly, although cfDNA VAF more closely approximates BM values in the technical cohort, this observation should be regarded as preliminary due to limited sample size. Second, the validation cohort (n = 40) was predominantly treated with HU, limiting generalizability to JAK inhibitor-treated populations. Additionally, we did not directly compare cfDNA and gDNA with paired BM samples in the validation cohort, which would have provided definitive confirmation of cfDNA’s superiority in reflecting BM clonal dynamics. Long-term clinical outcomes were not assessed, precluding correlation of cfDNA dynamics with survival or transformation risk.

Future studies should validate our observations, incorporating serial BM samples in larger cohorts, investigate the mechanistic basis for the lymphocyte percentage correlation, and evaluate cfDNA performance in the context of newer therapies such as ropeginterferon alfa-2b and novel JAK inhibitors. The recent demonstration that molecular response to IFN therapy correlates with improved clinical outcomes in MPN underscores the importance of optimizing molecular monitoring strategies (49). The potential for cfDNA to detect minimal residual disease and predict transformation risk also merits investigation. Prospective trials comparing outcomes in patients managed with cfDNA-guided versus gDNA-guided treatment decisions would provide definitive evidence for clinical implementation.

## 5 Conclusion

In conclusion, while constrained by sample size and specific treatment contexts, our study highlights the complementary utility of cfDNA and gDNA. Methylation-based deconvolution provides exploratory evidence for the BM contribution to cfDNA, while the CDX model demonstrates its capacity to serve as a superior surrogate for marrow clonal burden. Clinically, cfDNA offers higher sensitivity for capturing treatment-related clonal dynamics and emerging resistance, while gDNA is more informative for confirming treatment response. In addition, lymphocyte percentage emerges as a potential predictor of cfDNA performance, enabling a rational selection between the two analytes. Taken together, integrating both analytes into monitoring strategies provides a preliminary framework for optimizing MPN management that warrants further validation in larger, prospective cohorts.

## Data Availability

The EPIC methylation data generated in this study have been deposited in the Gene Expression Omnibus (GEO) under accession number GSE325660, and the dataset has now been released and is publicly accessible.

## Supplementary material

Supplementary Table 1 List of 582 CpG sites included in the reference methylation atlas.

Supplementary Table 2 Patient-level mutation and hematologic data of the technial cohort.

Supplementary Table 3 Patient-level mutation and hematologic data of the validation cohort.

Supplementary Table 4 Discordant mutations between gDNA and cfDNA in validation cohort.
