# Supplementary material for: Plasma cfDNA and peripheral blood gDNA provide complementary information for molecular monitoring in myeloproliferative neoplasms
Source: Front Oncol. 2026 Mar 27;16:1771587. doi: 10.3389/fonc.2026.1771587 (PMC13065645; doi:10.3389/fonc.2026.1771587)
Supplement: Supplementary file 5 [file DataSheet1.docx]

Supplementary Material

# Supplementary Methods

**Reference atlas construction**

Genome-wide methylation data for healthy bone marrow (BM; n=6, GSE124413) and whole peripheral blood (PB; n=6, GSE235717) were processed on the Illumina EPIC platform. CpG probes with missing values or low variability (variance <0.001 across samples) were removed. To identify BM and PB-specific CpG markers, methylation values were organized into a matrix **X** (CpGs × sample types). For each CpG i, row-wise normalization was performed as:$X_{i}^{'}=\frac{\boldsymbol{X}_{i}}{\sum_{j}^{d} \boldsymbol{X}_{i,j}}.$ The top **K**=100 hypermethylated CpGs for each tissue were selected based on the highest $\boldsymbol{X}_{\boldsymbol{i,j}}^{\mathbf{'}}$values, and top **K** hypomethylated CpGs were similarly identified using the inverted methylation matrix (1 − **X**). For each selected CpG, proximal CpG sites within ±50 bp were included to capture regional methylation structure. This procedure yielded a reference atlas of 582 CpGs spanning BM and PB (Supplementary Table1).

**Simulation of in-silico mixtures and model correction**

Independent BM and PB methylation datasets were used to generate in-silico mixtures at predefined proportions (0–10%, 50 replicates per condition). The deconvolution framework described by Moss et al. was applied to estimate tissue compositions. For each tissue type and mixture ratio, median predicted values, interquartile ranges, and Spearman correlations versus ground truth were computed. Systematic biases observed in BM and PB predictions were corrected using the fitted linear model: $Ycorrect=\frac{Y+|b|}{a}$. Corrected predictions were reassessed using Bland–Altman analysis, and the PB limit of quantification (LOQ) was determined. The corrected reference model was subsequently applied to patient cfDNA samples to quantify BM and PB-derived contributions.

# Supplementary Figures


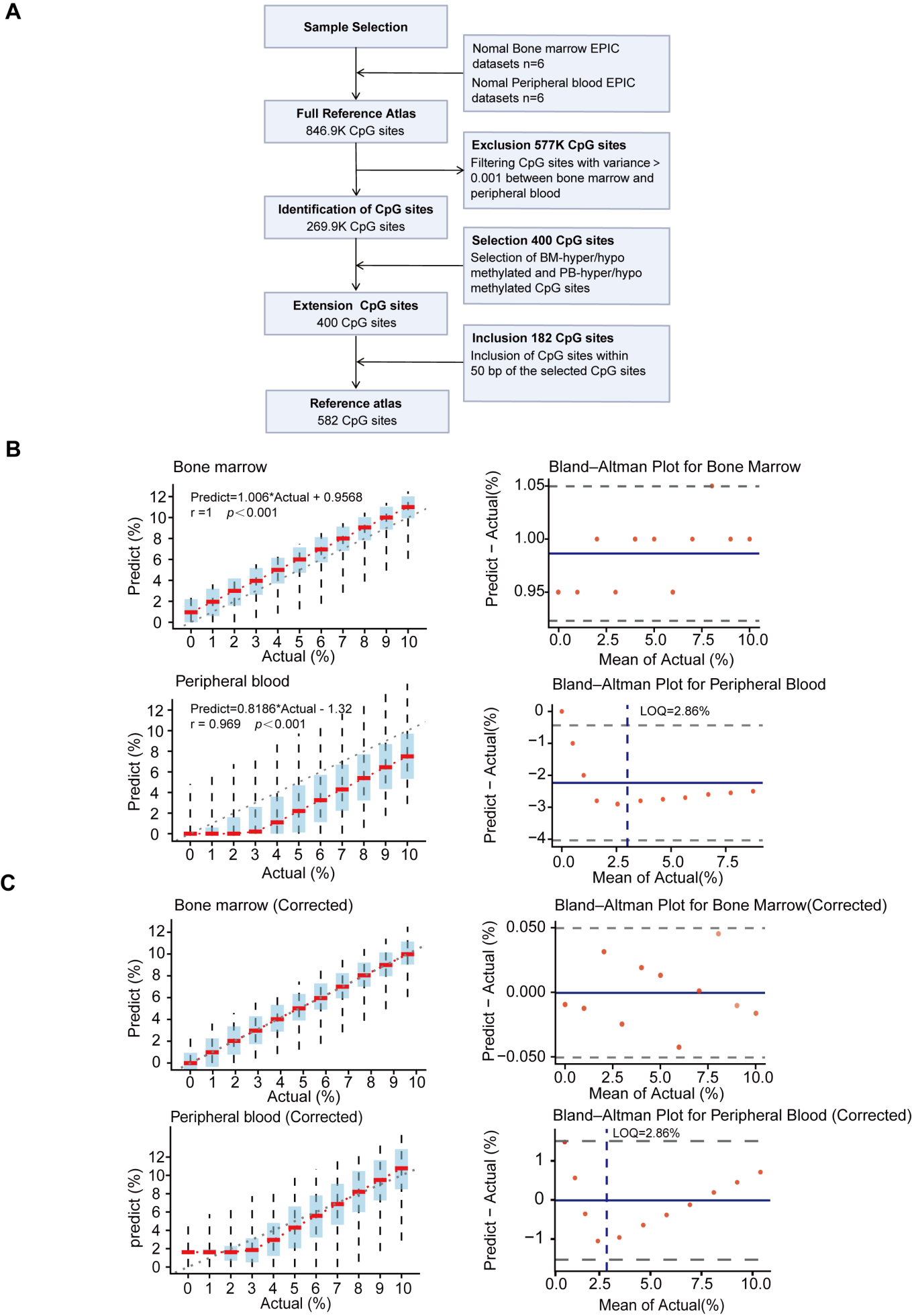


**Supplementary Figure 1.** **Construction and evaluation of the methylation atlas.**

(A) Workflow illustrating the construction of the BM and PB methylation reference atlas. CpG sites with detection *P* > 0.01. For each tissue type, the top 100 uniquely hypermethylated and 100 uniquely hypomethylated CpG sites were selected, followed by inclusion of neighboring CpGs within ±50 bp to generate a tissue-discriminatory feature set containing a total of 582 CpG sites.

(B) The methylome of each sample type was mixed in silico with the counterpart tissue (BM or PB, respectively) such that it contributed between 0% and 10% of total DNA at predefined intervals (x-axis) with 50 replicates for each mixture level. Deconvolution was then performed using the reference methylation atlas, and the predicted contribution of each sample type is shown on the y-axis. Results are presented as red horizontal bars (median) with a blue shaded area (interquartile range, 25th–75th percentile) and black vertical lines (9th–91st percentile). Bland–Altman analyses were performed to assess the agreement between predicted and actual proportions in the synthetic mixing experiments. For both BM and PB, the difference between predicted and actual values (y-axis) is plotted against their mean (x-axis). The solid blue line represents the mean bias, and the dashed grey lines indicate the limits of agreement (mean ± 1.96 SD). For PB, a blue line marks the limit of quantification (LOQ).

(C) In-silico simulation after linear correction, Bland–Altman analyses were repeated to evaluate residual bias.


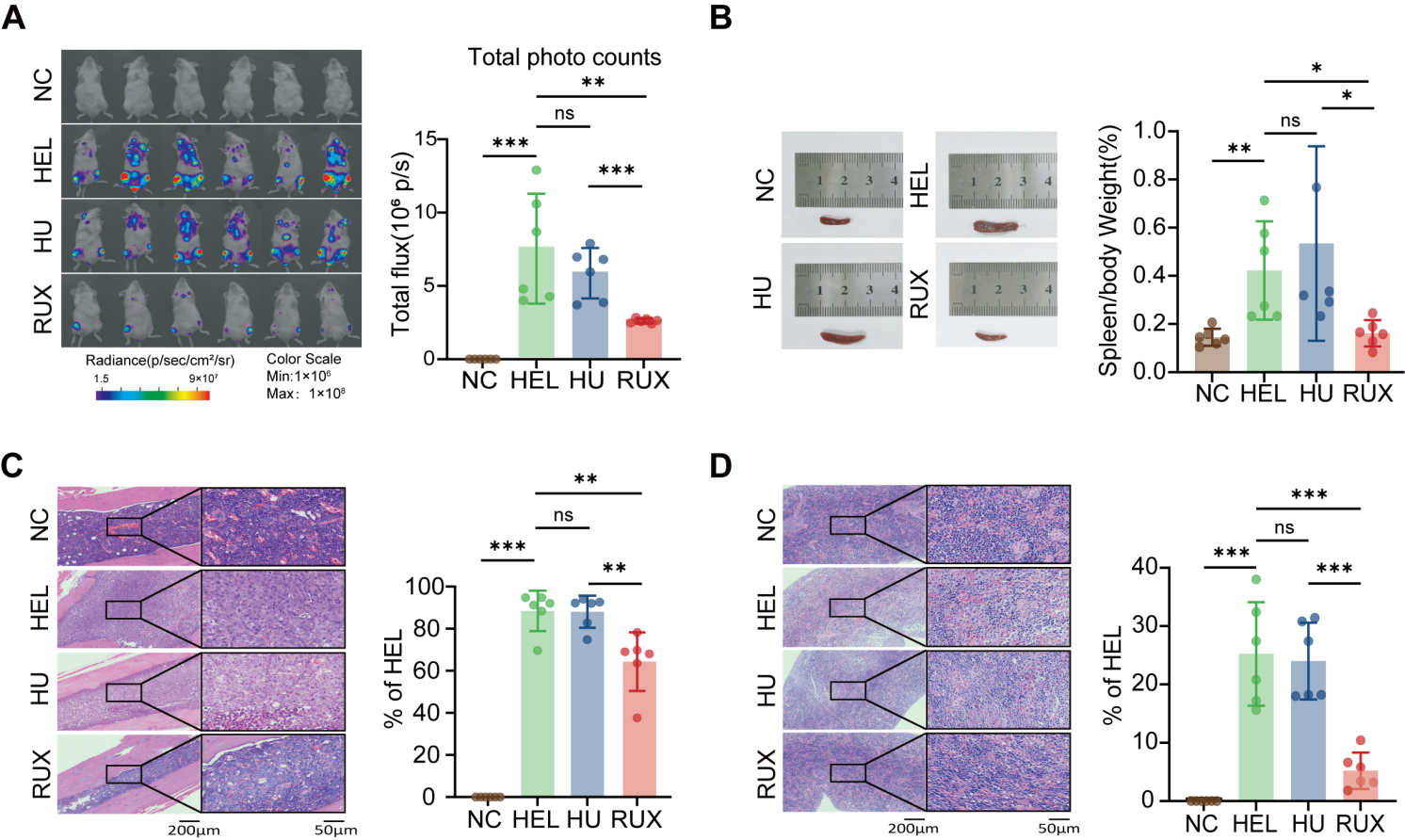


**Supplementary Figure 2. CDX model verification**

(A) Whole-body bioluminescence imaging at the end of treatment showing the distribution of HEL-derived signals in vivo. Bars show mean ± SD; ***P* < 0.01, *** *P* < 0.001 by independent-samples t-test

(B) Assessment of spleen morphology and spleen weight at necropsy. Bars show mean ± SD; **P* < 0.05, ** *P* < 0.01 by independent-samples t-test.

(C) Histologic analysis of bone marrow collected at terminal evaluation. Bars show mean ± SD; ***P* < 0.01, *** *P* < 0.001 by independent-samples t-test.

(D) Histologic analysis of spleen collected at terminal evaluation. Bars show mean ± SD, ****P* < 0.001 by independent-samples t-test.


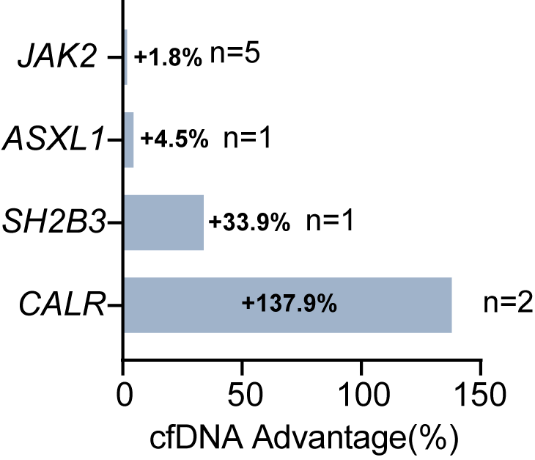


**Supplementary Figure 3. Gene-specific cfDNA advantage in the technical cohort**

Gene-specific analysis showing cfDNA advantages for *CALR* (137.9%, n=2), *SH2B3* (33.9%, n=1), *ASXL1* (4.5%, n=1), and *JAK2* (1.8%, n=5).


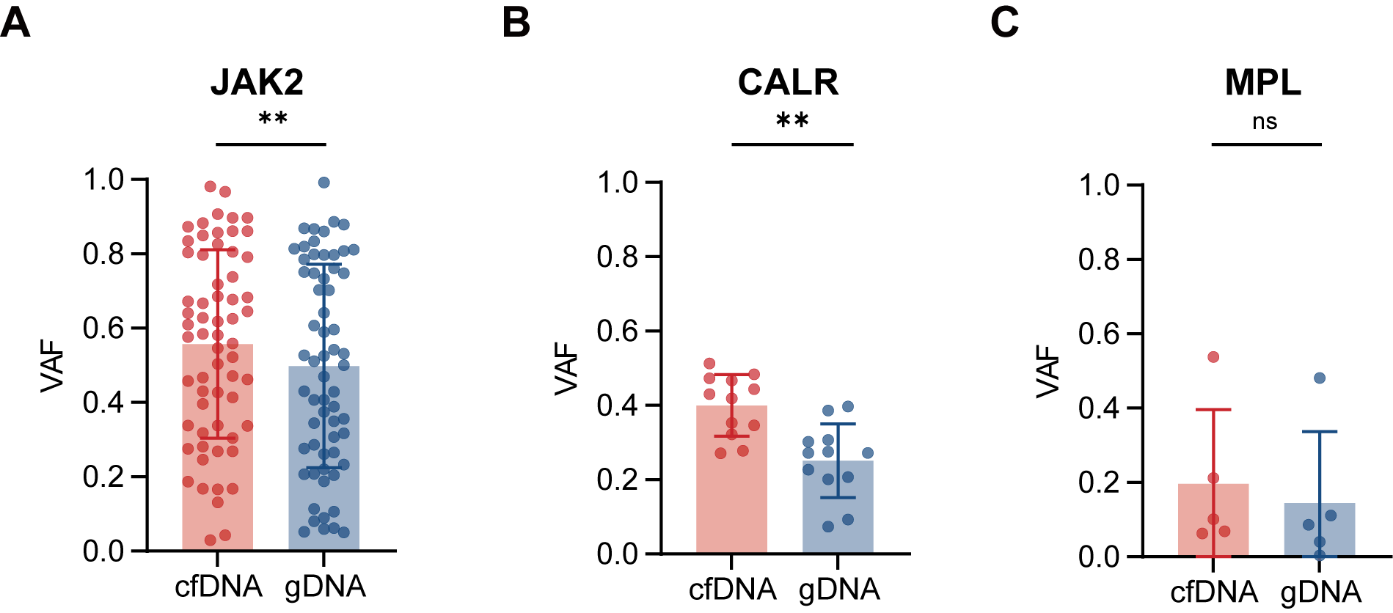


**Supplementary Figure 4. VAF comparison between cfDNA and PB gDNA** **across driver mutations in the validation cohort**

(A) VAF comparison of *JAK2* between cfDNA and PB gDNA. Bars show mean ± SD; ***P* < 0.01 by paired t-test.

(B) VAF comparison of *CALR* between cfDNA and PB gDNA. Bars show mean ± SD; ***P* < 0.01 by paired t-test.

(C) VAF comparison of *MPL* between cfDNA and PB gDNA. Bars show mean ± SD; *P*-value by paired t-test.


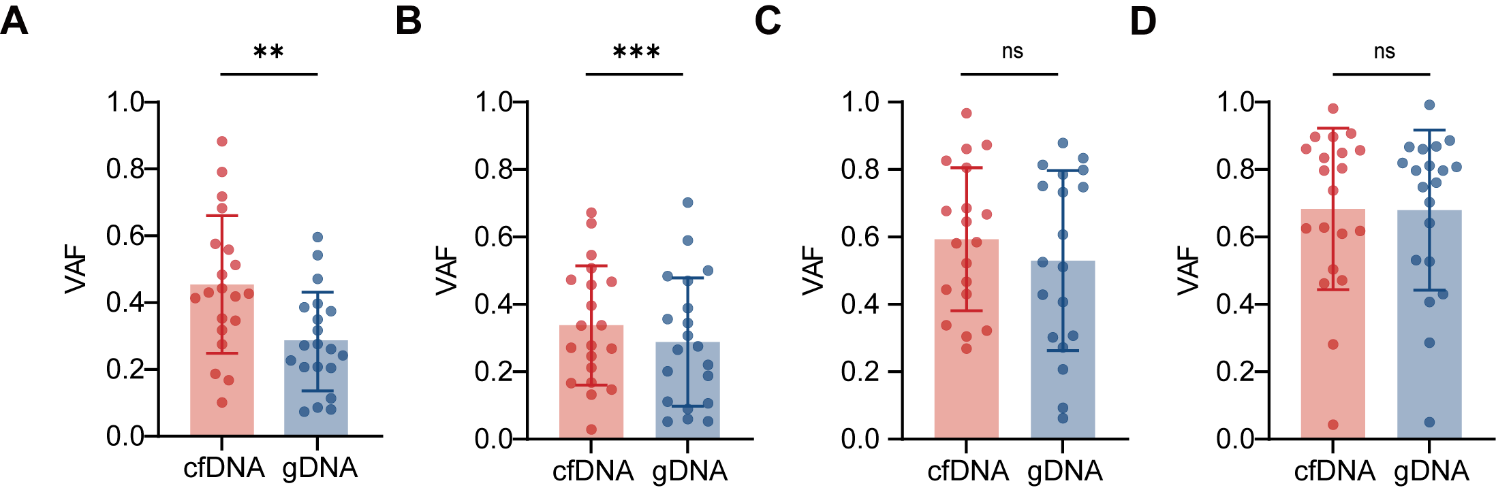


**Supplementary Figure 5. Comparative analysis of VAF measurements between cfDNA and PB gDNA stratified by WBC count quartiles.**

(A) Comparison of VAF measurements in the lowest WBC quartile (Q1). Bars show mean ± SD; ***P* < 0.01, by paired t-test.

(B) Comparison of VAF measurements in the second WBC quartile (Q2). Bars show mean ± SD; ****P* < 0.001, by paired t-test.

(C) Comparison of VAF measurements in the third WBC quartile (Q3). Bars show mean ± SD; *P*–value by paired t-test.

(D) Comparison of VAF measurements in the highest WBC quartile (Q4). Bars show mean ± SD; *P*–value by paired t-test.


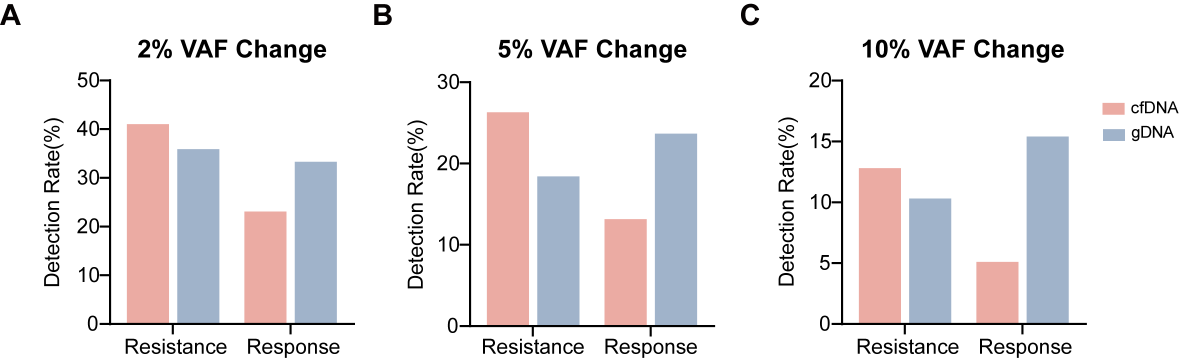


**Supplementary Figure 6. Classification of treatment response and resistance based on different VAF thresholds.**

(A) Treatment response and resistance classification using a 2% VAF threshold.

(B) Treatment response and resistance classification using a 5% VAF threshold.

(C) Treatment response and resistance classification using a 10% VAF threshold.


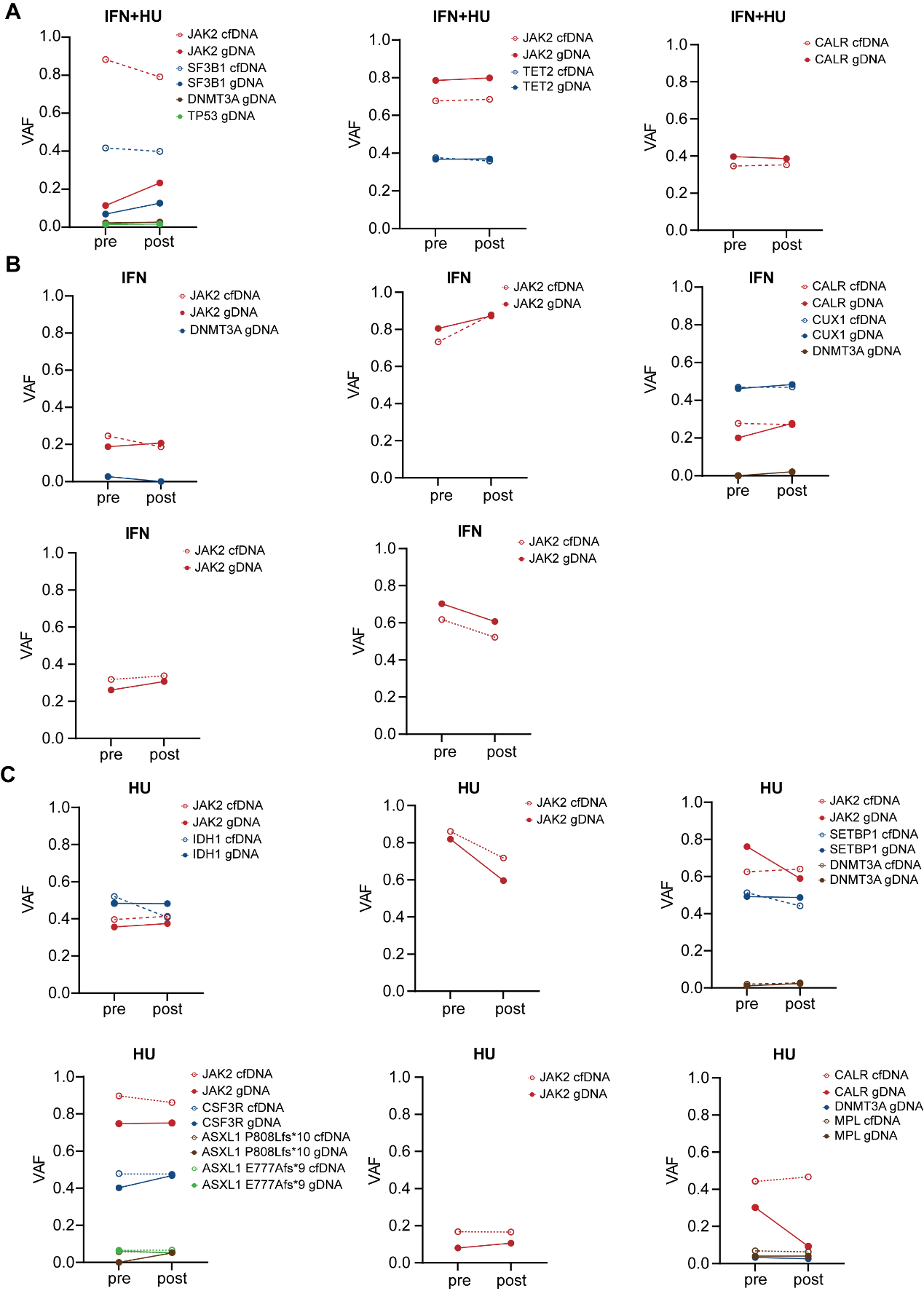


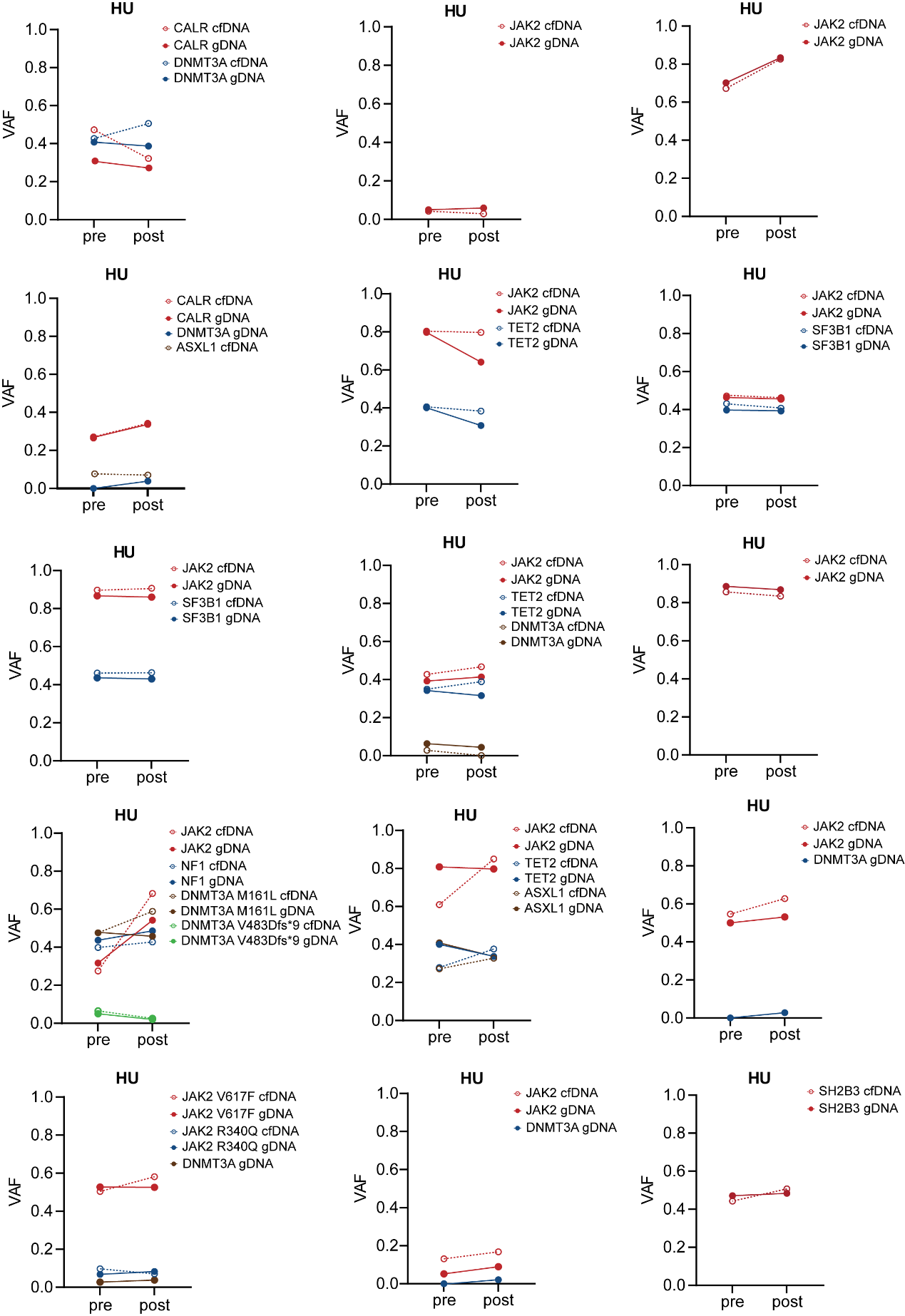
**
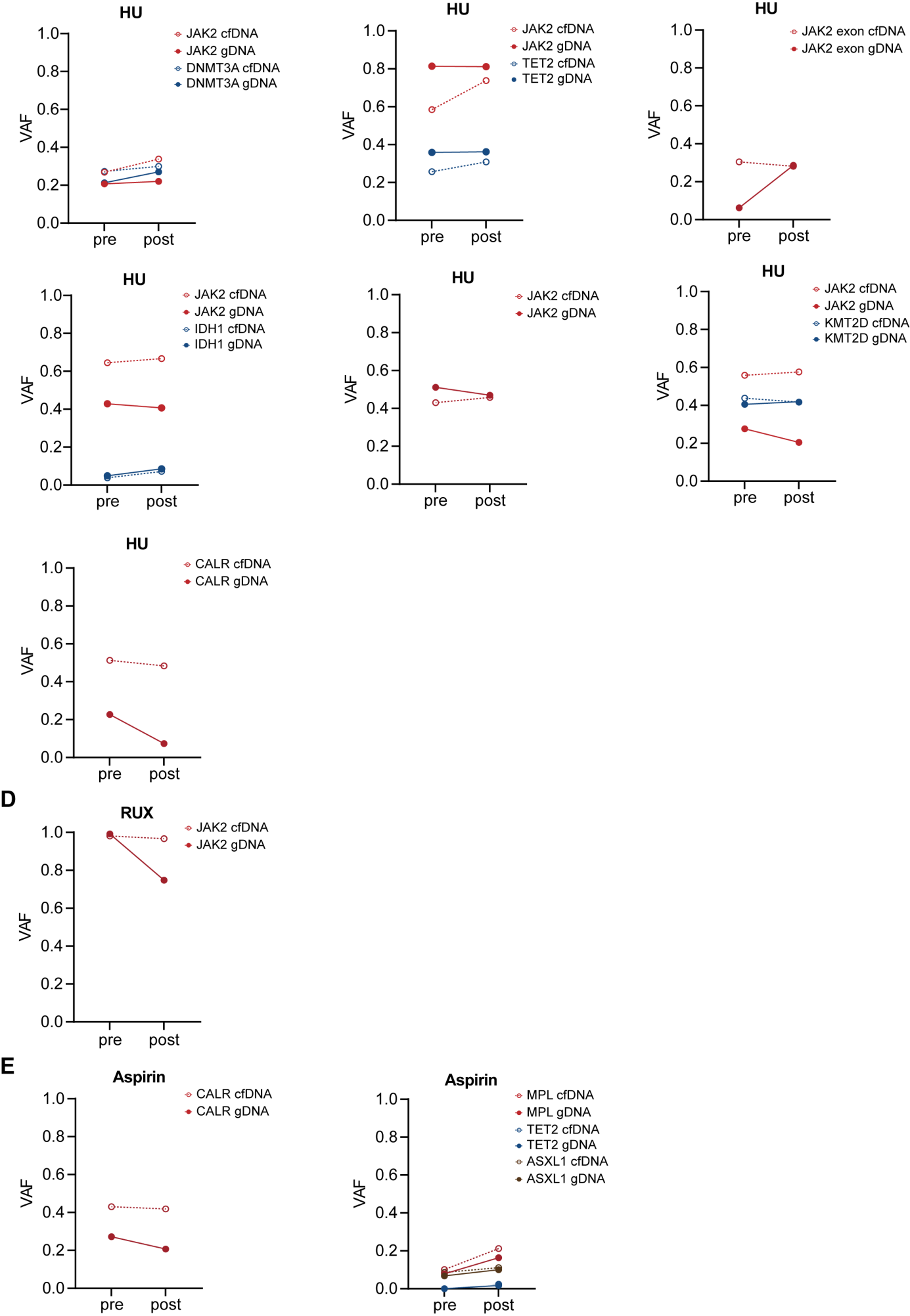
 Supplementary Figure 7. Individual patients’ clone evolution in different treatment groups.**

(A) Three patients receiving IFN combined HU therapy, dynamic changes of driver and non-driver mutation VAF measured by cfDNA (dotted lines) and gDNA (solid lines).

(B) Five patients receiving IFN therapy, dynamic changes of driver and non-driver mutation VAF measured by cfDNA (dotted lines) and gDNA (solid lines).

(C)Twenty-eight patients receiving HU therapy, dynamic changes of driver and non-driver mutation VAF measured by cfDNA (dotted lines) and gDNA (solid lines).

(D) One patient receiving RUX therapy, dynamic changes of driver and non-driver mutation VAF measured by cfDNA (dotted lines) and gDNA (solid lines).

(E)Two patients only receiving aspirin therapy, dynamic changes of driver and non-driver mutation VAF measured by cfDNA (dotted lines) and gDNA (solid lines).
